# Navigating Between Right, Wrong, and Relevant: The Use of Mathematical Modeling in Preclinical Decision Making

**DOI:** 10.3389/fphar.2022.860881

**Published:** 2022-04-12

**Authors:** Anna Kondic, Dean Bottino, John Harrold, Jeffrey D. Kearns, CJ Musante, Aleksandrs Odinecs, Saroja Ramanujan, Jangir Selimkhanov, Birgit Schoeberl

**Affiliations:** ^1^ Nektar Therapeutics, San Francisco, CA, United States; ^2^ Takeda Development Center Americas, Inc. (TDCA), Lexington, MA, United States; ^3^ Seagen Inc., South San Francisco, CA, United States; ^4^ Novartis Institutes for BioMedical Research Inc., Cambridge, MA, United States; ^5^ Pfizer Worldwide Research Development and Medical, Cambridge, MA, United States; ^6^ Genentech Inc., South San Francisco, CA, United States

**Keywords:** model-informed decision making, predictive modeling, translational modeling, modeling case studies, research and preclinical development

## Abstract

The goal of this mini-review is to summarize the collective experience of the authors for how modeling and simulation approaches have been used to inform various decision points from discovery to First-In-Human clinical trials. The article is divided into a high-level overview of the types of problems that are being aided by modeling and simulation approaches, followed by detailed case studies around drug design (Nektar Therapeutics, Genentech), feasibility analysis (Novartis Pharmaceuticals), improvement of preclinical drug design (Pfizer), and preclinical to clinical extrapolation (Merck, Takeda, and Amgen).

## 1 Introduction

The goal of this article is to provide a targeted perspective on how modeling and simulation approaches have been used to inform various decision points during the ‘R’ phase of Research and Development, namely from discovery to First-In-Human clinical trials. It is worth mentioning that the full adoption of modeling and simulation approaches in pharma has historically lagged compared to other industries, where products are routinely simulated even before being built. The reasons for that could largely be grouped in two main categories: technical and institutional. The first group is much easier to explain: lack of appropriate quantitative measurements and computational power to inform the development of adequate models was, in fact, true 20 years ago. However, this is much less of an issue today. The second category relates to biological complexity. Mathematical modeling and computer simulations have been an essential part of product development in just about every branch of science, engineering, and technology. The application of such approaches to reverse-engineer biology and to “design” novel therapies has been hindered by the lack of a pre-existing mathematical description of the broad range of biology involved and the common belief that the complexities of human health are too intractable to be addressed by computer models. The gradual change from resistance to acceptance by pharma and biotech companies has been aided by three factors: 1) success stories, such as the use of population pharmacokinetic/pharmacodynamic (PK/PD) models for dose selection or the widespread the use of physiologically-based PD (PBPK) models to assess drug-drug interactions in silico, 2) the wider availability of diverse data that are challenging to fully understand in the absence of integration into mathematical models, and 3) external pressure on the industry to accelerate development timelines and reduce potential late stage failures. All three factors have been recognized and supported by changes in regulatory policy. The FDA MIDD (Model-Informed-Drug-Development) program is a prime example of how regulators value the power of mathematical models and what they can bring to drug development. The creation of dedicated modeling functions within pharmaceutical and biotech companies has led to an increased investment and adoption of modeling approaches to inform decisions within R&D. While most of the investment has traditionally occurred in modeling of the clinical development stages, the insight that mathematical models can impact the design of novel therapeutics and allow us to anticipate the clinical experiences through early simulation of potential human scenarios have led to an increased investment in preclinical modeling and simulation activities.

To illustrate the value of such approaches in pre-clinical stages of R&D, our cross-institutional team of authors has aimed to provide here concrete examples where we have used quantitative approaches to impact decisions in these early stages. We decided that this approach, while more colloquial in nature, would be a nice complement to existing papers in the literature providing details around specific modeling methodology. We hope that the reader will find this collection of examples informative and thought-provoking. We have been privileged to be part of institutions and teams that worked on interesting and challenging problems. We have also been lucky to live in times of increased biological understanding and technology developments. But, most importantly, we live in times when society demands that our industry lives up to the promise of personalized medicine—understanding the etiology of disease for any given patient and finding solutions that will work for that patient. Albert Einstein once said: “Learn from yesterday, live for today, hope for tomorrow. The important thing is not to stop questioning”. He also famously said: “Everything should be made simple, but not simpler”. We hope that by cataloging our experiences, we provide simple examples that helps streamline future uses of models in preclinical drug development.

The organization of this paper is as follows. We begin by providing a high-level summary or enumeration for the types of problems in the preclinical stage of drug development that might benefit from quantitative approaches. Then we proceed to provide a more detailed description of a case study that illustrates an approach to such a problem from our experience in our various organizations. Finally, we finish with a few words of reflections and hopes for the future.

## 2 High-Level Summary of Decisions Benefiting From Quantitative Approaches

We work in a highly regulated industry in which development of new investigational medicines must comply with high standards to ensure that we understand the anticipated safety of the proposed interventions prior to bringing them into the clinic. Furthermore, the high cost of R&D necessitates continuous consideration of the probability of clinical success with respect to risk-benefit tradeoffs. Per FDA guidance (https://www.fda.gov/patients/drug-development-process/step-2-preclinical-research), the first two steps of the drug development process are 1) discovery and development, and 2) preclinical research. A key objective that arises in these stages is to identify a promising therapeutic target that can help alter the course of human disease or treat symptoms. We then screen among the different possible drug candidates to select the most promising candidate based on the interplay between several factors including: pharmacological activity for potential efficacy; Absorption, Distribution, Metabolism, and Excretion (ADME), and pharmacokinetic (PK) properties; side effects (toxicity); and how a particular modality compares with existing treatments. It is necessary to address these objectives while balancing resource constraints with the goal to progress further potentially promising programs while ending those that are less promising ones as early as possible. There are ample opportunities for quantitative approaches to be used to aid the decision making at this stage.

Our list of decisions supported by modeling in the discovery space is presented below:

1) Target or modality assessment:
  a) Feasibility assessment
  b) Competitive evaluation
  c) Repurposing of existing targets and molecules
2) Rational Drug Design and Compound Selection
  a) Desired drug property optimization
  b) Molecule generation and selection
3) Preclinical study design
4) Toxicology assessment (organ-specific)
5) Interspecies translation and clinical regimen design
  a) Clinical Study Design: PKPD, safety, efficacy
  b) Animal rule for translation-based approval

Before going to the specifics of the case studies, we wanted to acknowledge that they do not cover all aspects in the list above. For example, multiple publications cover topic (Brown et al., 2003) with examples of renal (Thomas, 2019), hepatic (Watkins, 2020), and cardiac toxicities (Amuzescu et al., 2021). In addition, while modeling can be a useful tool for the repurposing of existing molecules in new diseases, this topic is not covered in this paper [for published considerations on the topic the reader should consider (Pushpakom et al., 2019; Gozzo et al., 2020; Verbaanderd et al., 2020)].

## 3 Detailed Case Studies

### 3.1 Novartis: Novel Modality and Feasibility Analysis

Novartis regularly applies modeling and simulation to assess the potential of new therapeutic concepts at early stages of drug discovery to inform Go/No-Go decisions and therapeutic design. Here, we describe a model-informed molecular design exploration and feasibility assessment of a theoretical antibody intended to treat obesity-related disorders. Obesity is becoming increasingly common, and the available treatment options do not fully address this problem (Mullican and Rangwala, 2018; Tsai et al., 2018). The therapeutic potential of GFRAL agonism with GDF15 has been demonstrated preclinically with multiple approaches. Mice and monkeys on a high-fat diet treated with either AAVhu-GDF15, recombinant GDF15, or a scFc-GDF15 fusion lost about 10–24% of their body weight over 5–6 weeks and showed reductions in key metabolic parameters (Xiong et al., 2018). However, the use of recombinant GDF15 as a therapeutic is limited by its short serum half-life of less than 3 h in human (Zorzi et al., 2019).

**FIGURE 1:**
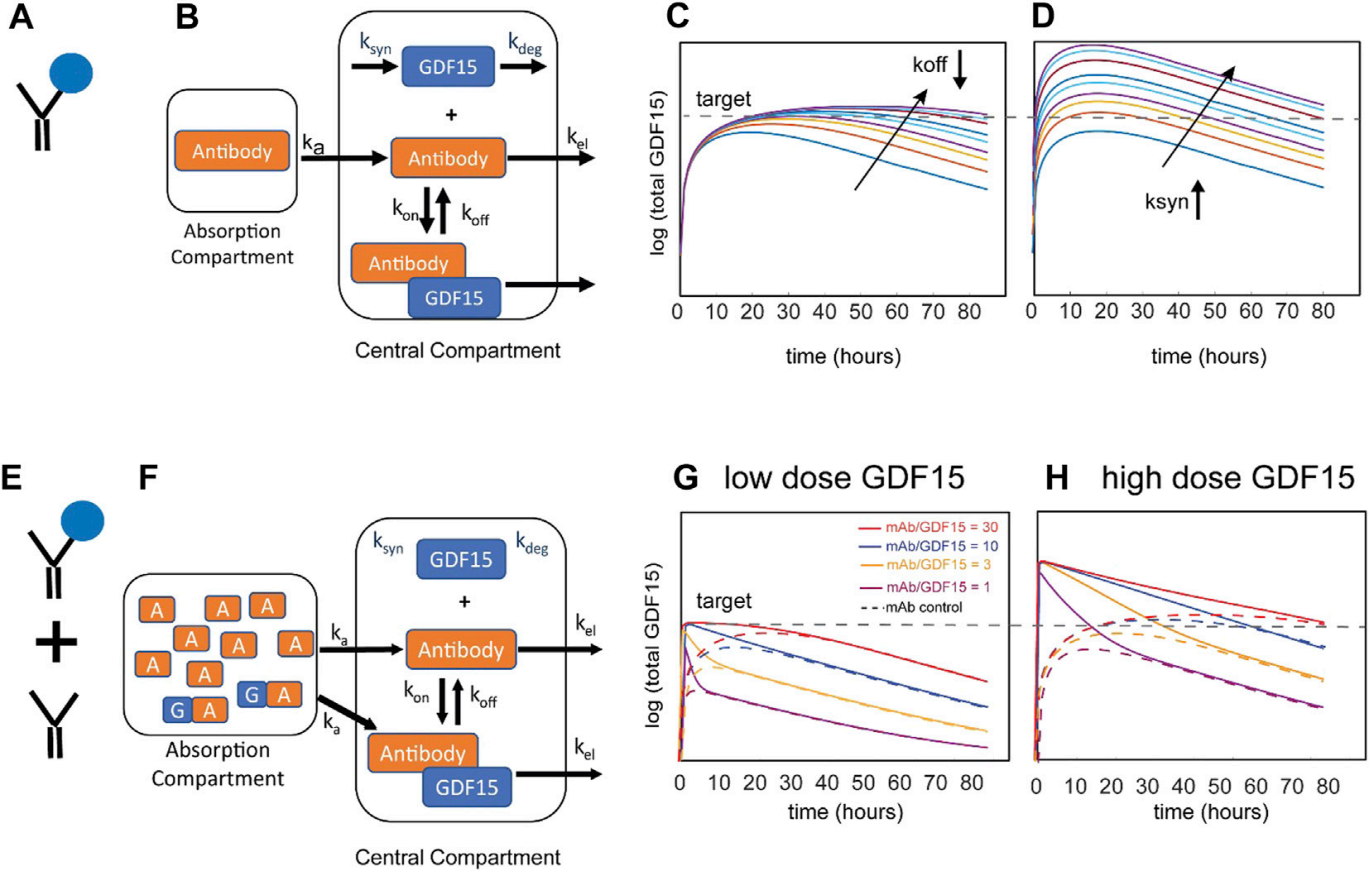
Schematic of the model for the Novartis case study and resulting simulations. Stabilization of endogenous GDF15 ligand via binding to therapeutic antibody **(A)** can be described with a one-compartment model **(B)**. Results of local parameter scans for increasing the stability of the GDF15:antibody complex **(C)** and increasing the pool of endogenous GDF15 **(D)**. The arrows represent rate constant modifiers from 1ȕ to 1/25ȕ (decreasing k_off_) or 1ȕ to 25ȕ (increasing ksyn), respectively. Administration of a mixture of stabilizing therapeutic antibody and recombinant GDF15 **(E)** can be described by the same one-compartment model **(F)**. Simulations of different mixture compositions with a low **(G)** or high **(H)** dose of exogenous GDF15. Shown in the solid lines are four ratios of antibody to GDF15 (30:1, 10:1, 3:1, 1:1 or equimolar). The stippled lines represent an antibody-only control for each of the four compositions.

In this example, the therapeutic antibody was proposed to bind to endogenous GDF15 to extend its half-life (Figure 1A) as an alternative to exogenous GDF15 approaches. The mechanistic hypothesis is that pharmacological stabilization of GDF15 with a non-antagonist antibody should increase circulating levels and thereby drive sustained GFRAL signaling, reduction in food intake, and weight loss. The Novartis team employed a small mechanistic PK/PD model for subcutaneous administration (Figure 1B) with a structure similar to published models of antibody-ligand traps (Davda and Hansen, 2010). Standard monoclonal antibody (mAb) PK parameters for cynomolgus monkeys and human were assumed. The drug PK, dosing regimen, affinity of the antibody:GDF15 complex, and patient-to-patient variability of baseline GDF15 levels (Brown et al., 2003) were explored to maximize the amount of circulating GDF15:mAb and to assess whether sufficiently high total GDF15 concentrations can be achieved.

Representative simulation results in Figure 1C show the impact of binding affinity on total GDF15 (by decreasing the antibody dissociation constant k_off_ at a constant dose). The simulations show that increasing the binding affinity does not meaningfully increase GDF15 Cmax but instead extends the time above threshold. This system behavior can be explained by the pool of total GDF15 quickly saturating despite free antibody being in excess. The GDF15 synthesis rate is the most sensitive parameter and predicted to increase GDF15 Cmax and time over threshold (Figure 1D). Subsequent consideration of synthesis rates derived from baseline GDF15 levels in patients revealed an increase of up to ∼100-fold (data not shown), suggesting that this concept may not be viable for most patients to achieve the 100-to-1000-fold increase identified as an efficacious threshold for the scFv-GDF15 fusion (Xiong et al., 2018).

The modeling analysis was extended to consider an alternative therapeutic approach that is less dependent on patient GDF15 levels: a mixture of free antibody and antibody pre-complexed with recombinant GDF15 (Figure 1E). This mixture allows the administration of recombinant GDF15 in excess over endogenous GDF15 levels and thus decouples the therapeutic from patient-specific levels of baseline GDF15. The same PK/PD model with different initial conditions was used to explore different ratios (Figure 1F). Since the antibody dose and amount of recombinant GDF15 can be modulated, it is theoretically possible to achieve much higher Cmax with no time delay and to “control” the time over threshold. In Figure 1G the impact of a low dose of GDF15 is shown. Looking at the total GDF15 concentration time course, a 1:1 ratio of mAb and GDF15 results in a sharp peak and behaves over time similarly to the mAb control that binds endogenous GDF15. As the ratio of mAb to GDF15 increases, the concentration time course of total GDF15 can be modulated to achieve sustained GDF15 levels. Figure 1H demonstrates that at a high dose of GDF15 precomplexed with antibody the time over threshold can now be extended from hours to weeks. To summarize, the amount of GDF15 and the mAb to GDF15 ratio allows for a lot of flexibility to modulate the shape of the total GDF15 concentration-time course. As a research tool, it allows to gain a deep understanding of what pharmacokinetic features like Cmax or time over threshold drive weight loss which ultimately informs the design of an optimized therapeutic.

### 3.2 Nektar Therapeutics: Rational Drug Design

**FIGURE 2:**
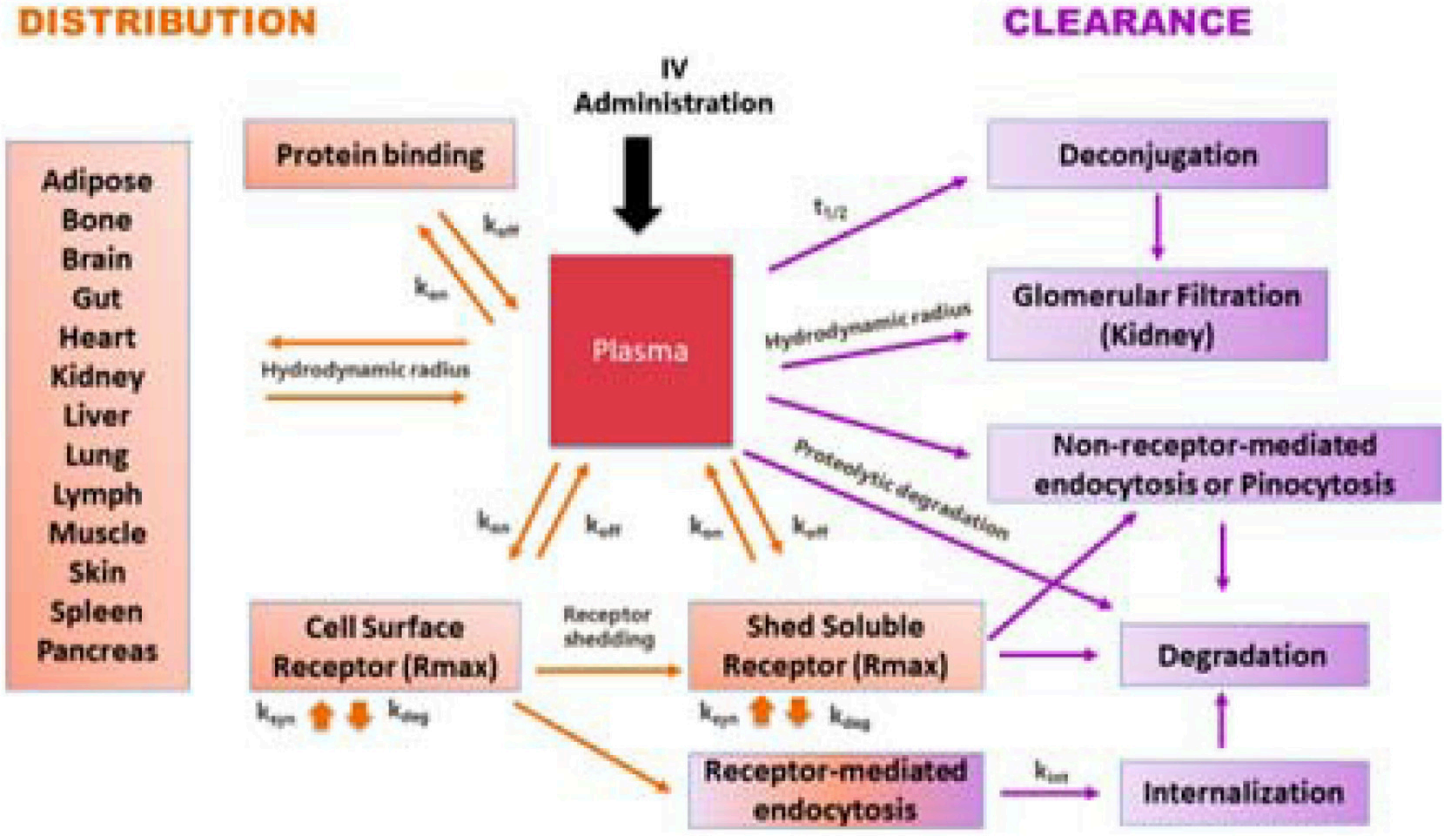
A schematic representation of a general modeling framework incorporating key processed involved in the disposition and clearance of PEGylated cytokines.

To systematically address questions in its portfolio of PEGylated cytokines (PEG = polyethylene glycol), Nektar created a predictive modeling platform in Simcyp™ PBPK Simulator (Certara, Inc., Princeton, New Jersey, USA) based on first principles. The PBPK modeling module for protein- based therapeutics in Simcyp™ is well established and continues to evolve by integrating new information. Its goal is to predict disposition and clearance for protein-based therapeutics. Figure 2 provides a brief overview of the processes impacting the disposition and clearance of large molecules and can be adapted for individual programs and molecules. This is important, because the framework is no longer just a system of equations but contains parameter values that have been derived based on experimental data. As such, the model has become an integrated database that can b used to guide experiments and aid dose, dosing regimen, and candidate selection.

Distribution to tissues is governed by permeability, partition, and binding to cell surface receptors. Key pathways contributing to clearance include glomerular filtration, pinocytosis, degradation, and internalization of receptor-bound molecules, (depending on the conjugation chemistry) release of PEG molecules. Understanding the physical-chemical characteristics, receptor binding kinetics (association and disassociation constants k_on_ and k_off_) and fate of receptor complex are important for development of a predictive model.

A PEGylated cytokine has larger hydrodynamic radius than its parent molecule. Experimental measurement of hydrodynamic radius during the drug design stage is not always practical. Therefore, we developed an artificial neuronal network model to estimate the hydrodynamic radius of a PEGylated cytokine based on the PEG molecular weight, the protein molecular weight, the PEGylated cytokine molecular weight, and the percent of PEG in the PEGylated cytokine. During the drug development stage, techniques such as Dynamic Light Scattering can be used to experimentally determine the hydrodynamic radius. Knowing the hydrodynamic radius of a PEGylated cytokine conjugate allowed us to simulate the amount of PEGylated conjugate eliminated by glomerular filtration. The biologics module in Simcyp™ is based on the two-pore theory; a cut off for glomerular filtration to molecules with a hydrodynamic radius larger than 6 nm. With this restriction, molecules with higher hydrodynamic radius, such as cytokines with molecular weight in a range of 10–30 kDa PEGylated with 60 kDa PEG or 50 kDa cytokine dimers, trimers, and tetramers PEGylated with 40 kDa PEG are not expected to be eliminated by glomerular filtration.

In addition to glomerular filtration, target-mediated drug disposition (TMDD) contributes to elimination of all cytokines. Standard receptor binding parameters such as k_on_ and k_off_ can be experimentally determined *in vitro*. The maximum achievable binding can be assumed to be the same for a given cytokine, whether PEGylated or not; it can be estimated using published PK profiles and the binding properties for the cytokine of interest. After a PEGylated cytokine binds to its receptor, the drug-target complex has two pathways: some molecules will dissociate, and some will undergo subsequent internalization and endocytosis. The receptors for some cytokines, such as IL-1, IL-2, and TNF-alpha, are shed, and interactions with the soluble receptors need to be considered. The Simcyp™ biologics module can be used to implement the interaction of cytokines with soluble receptors, which can result in dissociation or degradation.

In instances when the PEG-conjugates exceed the glomerular filtration cutoff value, the relative contribution of non-receptor-mediated endocytosis or pinocytosis in their elimination increases. The reference CL through pinocytosis is ∼0.01 L/h, which is the reported CL value for Cimzia® (Cimzia Full prescribing information, 2017). Cimzia® is a humanized antigen-binding 50 kDa fragment (Fab’) of a monoclonal antibody that has been conjugated to a 40 kDa PEG. The hydrodynamic radius of this 90 kDa PEG-conjugate prevents glomerular filtration and TMDD was reported not to contribute to its clearance. Hence, the observed clearance values for Cimzia® provide a good estimate for the magnitude of clearance by pinocytosis, a constant, non-saturable process. Alterative methodologies for estimating rate of pinocytosis (e.g., expanding the *in vitro* pinocytosis rate in endothelial cells to the whole body), generate values within 3 times the Cimzia® reference value. For PEGylated molecules with conjugation chemistries that release PEG molecules *in vivo*, PK profile predictions require estimation of the PEG release rate, which can be directly measured or obtained by fitting a parameter to experimental data.

In summary, the modeling platform used by Nektar Therapeutics considers key molecular attributes such as hydrodynamic volume and receptor binding kinetics. This platform can be used to evaluate the impact of different PEGylation strategies on PK, thus contributing to rational drug design and informing decisions in the preclinical stages of R8D.

### 3.3 Genentech: Molecule Design and Compound Selection

**FIGURE 3:**
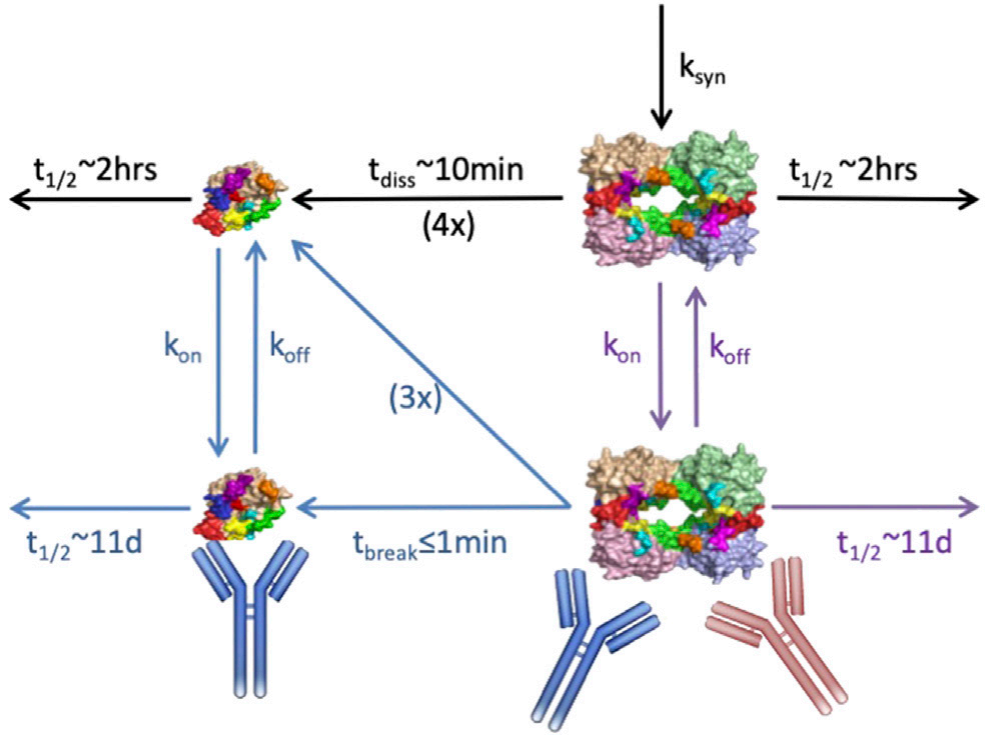
Schematic of anti-tryptase PKPD model with lung compartment shown. Mechanisms represented include: tryptase tetramer secretion in the lung, physiological dissociation of tetramer to four monomers, antibody binding to/dissociation from the monomeric, and tetrameric forms, as appropriate, and binding induced disruption *vs.* stabilization of the tetramer. Black arrows represent physiological mechanisms; red antibody/arrows pertain to tetramer-selective stabilizing molecule; blue antibody/arrows pertain to destabilizing molecule; purple arrows pertain to both. Standard two-compartment systemic/peripheral nonspecific PK augmented by binding to monomer in the serum, and drug partitioning to lung were also included in the model but are not shown.

Modeling can have meaningful impact early in the R&D process when used to compare alternate mechanisms for antagonizing a target to inform molecule generation and selection. In one such example, Genentech was exploring an antibody-based approach to targeting the protease tryptase in the lung for treatment of asthma and allergic airway disease. Tryptase is assembled into an active tetrameric molecule within acidic granules of mast cells and released in this active tetrameric form during degranulation. At extracellular pH, the tetramer dissociates relatively quickly into four inactive but longer-lived monomers; the combined effects of 4:1 stoichiometry and increased stability render the inactive monomer more abundant physiologically than the active tetramer. The molecule team had developed a destabilizing antibody that bound and rapidly disassembled the tetramer, but also bound the monomer with similar binding kinetics. Due to concerns that unproductive binding to the more abundant monomeric form would reduce drug availability, the team was also generating tetramer-selective antibodies, although these formed stabilizing complexes with the tetramer, inhibiting their physiological dissociation. Thus, a mechanistic PKPD model was developed and applied to quantitatively compare the ability molecules with these different Mode of Action (MoA) to neutralize tryptase activity in the lung (Figure 3).

The modeling results indicated a clear advantage of the destabilizing antibody under various scenarios for systemic and lung tryptase concentrations, despite the molecule’s nonproductive binding to inactive monomer (Chen et al., 2020). The quantitative simulations highlighted that tetramer destabilization leads to efficient reduction in the active species across the dose-regimens and concentration scenarios evaluated, superior to that achieved by the stabilizing antibody. Further, because binding-induced destabilization occurs even faster than antibody dissociation, a relatively fast dissociation rate (i.e., a higher k_off_ and K_D_) can reduce unproductive engagement of drug with monomer without compromising tetramer inhibition. For the stabilizing antibody however, the model suggested >10x lower K_D_ molecules would be needed for near-comparable inhibition due to the need to continuously engage target. These simulations drove the decision to focus on development of the destabilizing antibody without further affinity improvements, saving significant time and money on antibody campaigns and optimization. The molecule was advanced and is now under clinical evaluation. Notably, the initial model structure also was expanded in subsequent PKPD efforts to capture nonhuman primate data and to then project and interpret clinical PKPD, thus enabling integration of knowledge and data alongside development, and providing value beyond its initial application to molecule design/selection.

### 3.4 Pfizer: Improvement of Preclinical Study Design for Obesity Target Using Model Informed Drug Development

Preclinical program decisions in drug discovery often rely on results from a set of key studies. In the following example, Pfizer illustrated how modeling and simulation can help increase confidence in conclusions that are based on results coming from such studies by informing their design.

The discovery and development of novel therapies for the treatment of obesity is challenging, often due to lack of clinical efficacy. A key to bringing the best possible anti-obesity therapies to the clinic is based on effective preclinical efficacy evaluation of anti-obesity targets. This evaluation is dependent on the understanding of the inter- and intra-animal variability of key endpoints, such as food intake and body composition, to aid in study design, and the proper interpretation of results. To address this, the team leveraged a model-based power analysis to propose guidelines for endpoint selection and study size to inform *in vivo* preclinical study design for anti-obesity programs.

Specifically, Selimkhanov et al. (2017) fit a published physiologically based (PB) model of energy balance (Guo and Hall, 2009; Guo and Hall, 2011) that describes the feedbacks and interrelationships between efficacy endpoints typically measured in weight loss studies to individual C57BL/6 mouse’s longitudinal data (Guo and Hall, 2009; Guo and Hall, 2011). The resulting statistical model described intra and inter subject variability that could be observed in a typical mouse study as well as the response of key endpoints to changes in metabolic energy balance. The statistical model was then used to simulate a typical study design with a hypothetical anorectic agent in order to estimate various endpoint effect sizes and variances. Using model-predicted effect sizes and variances, the team was then able to calculate the number of animals necessary to achieve sufficient statistical power for different endpoints.

The results of the analysis indicated that food intake variability is driven primarily by day-to-day intra-animal variability, whereas body weight and fat mass variability were driven primarily by differences between animals, important factors to consider in endpoint selection. Moreover, the analysis highlighted the need for caution when interpreting results from small preclinical studies that are not statistically powered for a given endpoint. As an example, in a simulated food intake reduction study powered to detect a change in body weight, the team found that the study also was sufficiently powered to detect a change in cumulative food intake; however, the study was underpowered to detect changes in other common endpoints, such as fat mass, fat- free mass, and single day food intake. In summary, model-based approaches such as this may be utilized to inform preclinical study design parameters, such as sample size and endpoint selection, as well as to aid in the proper interpretation of results for improved preclinical efficacy evaluations.

### 3.5 Translational Modeling in Oncology

The goal in this class of problems is to inform a possible dosing regimen in the clinic using data that typically comes from tumor-bearing or syngeneic mice. One of the goals is to build these models in such a way that they can continue incorporating new data, decreasing the uncertainty of the model predictions.

#### 3.5.1 Merck

In the case of dinacyclib, a selective CDK1,4 small molecule inhibitor, different doses were studied in a Phase 1 setting yielding information on the pharmacokinetics, pharmacodynamics and safety of the drug, as well as the tolerability of the drug. there was a clear picture for dose limiting toxicity, based on the phase 1 clinical data (Nemunaitis et al., 2013; Mita et al., 2017). Information on the shape of dose response curves in tumor-bearing mice for different tumor types was also available (Mehrara et al., 2007). The task was to use this information and combine it with the data being generated from a satellite PK study (Booher et al., 2014) to determine the width the therapeutic window would be for this molecule.

1) The translation on the PK side was done by using a hybrid PBPK model with a tumor compartment using data from both preclinical and clinical studies, while accounting for any differences in plasma protein binding.
2) On the PD side, the dataset consisted of 1) tumor growth curves for the different tumor types and corresponding dose levels for tumor bearing mice and 2) an epidemiological dataset that described the observed tumor doubling times for different tumor types in patients, reporting on the variability. Starting from the doses inducing dose limiting toxicities (DLT-s), the translational model was then used to simulate dose-exposure-response scenarios providing a simple guide for further clinical investigations.

#### 3.5.2 Takeda

A similar approach was taken by Takeda (Bottino et al., 2019) where a methodology was developed to determine the most appropriate dose and dosing regimen for novel oncology combination, consisting of two small molecules, inhibiting the PI3Kα, and mTOR pathways. As above, this modeling framework utilizes preclinical anti-tumor activity data and phase 1 clinical toxicity data, but for two, rather than a single molecule. The principal methodology, set up as a two-dimensional constrained optimization problem can be described in the following steps:

• Modeled observed antitumor activity as a function of drug concentration. All doses were converted to human-equivalent free fraction-corrected exposures (as in the Merck example).
• This methodology depends on clinical toxicity data, using bivariate logistic regression. One can make a point that quantitative systems toxicology models can be used to extrapolate preclinical data should this information be not available. Maximum tolerated exposure (MTE) curve in this case was defined as the set of exposures predicted to result in 25% probability of DLT.
• The MTE curve was then overlaid on the preclinically determined efficacy surface to see which tolerable concentration pair maximized the anti-tumor activity and doses that led to the optimal concentrations were back calculated.

### 3.6 Amgen: Translational Modeling - Application of Animal Rule

For some indications it is not feasible or ethical to conduct clinical trials to evaluate the efficacy of therapeutics. Development of therapeutic interventions for these indications can utilize the Animal Rule where efficacy is established in a well-controlled animal study using an animal model that best represents the indication of interest (US Food and Drug Administration, 2019). These pivotal studies will establish the efficacy of the therapeutic as well as the exposures and other metrics associated with that level of efficacy. Because it is not possible to verify the efficacy of the therapy in human subjects, it is important to establish that the human dosing is likely to achieve or exceed efficacy metrics in the preclinical species.

The approval of granulocyte colony stimulating factor treatments (G-CSF), filgrastim and pegfilgrastim, for the treatment of acute radiation syndrome (ARS) provides an example of the challenges associated with combining preclinical and clinical data together to identify the proper dosing under the Animal Rule. ARS results from acuate exposure to high doses of radiation leading to myelosuppression. As a result, individuals develop neutropenia and are exposed to opportunistic infections that can lead to increased mortality. Treatment with G-CSF can stimulate the production of granulocytes and reduce the duration of neutropenia after a hematopoietic injury. Two pivotal studies were sponsored by the National Institute of Allergy and Infectious Disease and conducted by the University of Maryland (Farese et al., 2013; Hankey et al., 2015). These establish the reduced duration of neutropenia and survival benefit of G-CSF treatment in non-human primates (NHP) exposed to lethal amounts of radiation. Because of their historic use in the treatment of chemotherapy induced neutropenia (CIN) there is substantial clinical data relating G-CSF treatment to exposures and neutrophil response in humans.

A series of models were developed to predict the effects of radiation and potential benefits G-CSF treatment on survival in humans. PK and ANC response data from healthy volunteers and patients (adults and pediatrics) with CIN were used to develop a mechanistic model. This accurately characterized the interplay of target-mediated disposition of both filgrastim and pegfilgrastim and the stimulation of ANC production in response to treatment (Melhem et al., 2018). This model allowed for the characterization of the underlying dynamics of granulocyte homeostasis as well as the impact of neutrophils on G-CSF PK. Two separate models were developed in parallel from the pivotal ARS NHP studies (Harrold et al., 2020a). The first model characterized granulopoiesis and radiation injury in NHPs. This effort utilized the same structural model of granulopoiesis from the human model. Next a time to event model was used to predict overall survival (OS) using the observed ANC profiles. Next these models were combined: Granulopoiesis and the disposition of filgrastim and pegfilgrastim from the human model was merged with the models of radiation injury and survival benefit in NHPs (Harrold et al., 2020b). The resulting model was calibrated using historical survival data in humans exposed to radiation to characterize the untreated response. Simulations were then used to evaluate the potential survival benefits of different G-CSF treatment regimens and the impact of delaying treatment.

## 4 Concluding Remarks

In this manuscript, we have detailed illustrative case studies from our experience that highlight how modeling and simulation is used to inform decision making in discovery and preclinical development. These examples only scratch the surface of this evolving modeling landscape which includes additional categories beyond the case studies here, for example, computational chemistry and structural biology, systems toxicology, and more. Nevertheless, there are some important high-level learnings that apply across various model applications:

1) Collaborative efforts between modelers and experimental scientists are key to creation of pragmatic models to influence decisions.
2) Models should come with clearly stated assumptions and relevant context of use.
3) Inaccurate model prediction from a well-designed and developed model should not be interpreted as an error, but rather an indication of a key knowledge gap.
4) Building models as integrated knowledge frameworks usually pays dividends to answer more than one question and inform development of multiple therapies in the portfolio.

In a field with continuously evolving technologies, data, and knowledge, we hope that the future will bring many more examples of impactful decision making from industry and academia, consortia efforts and government research.

## Data Availability

Publicly available datasets were analyzed in this study. This data can be found here: na.
